# A Liquid–Liquid Phase Separation-Related Index Associate with Biochemical Recurrence and Tumor Immune Environment of Prostate Cancer Patients

**DOI:** 10.3390/ijms24065515

**Published:** 2023-03-14

**Authors:** Qi You, Jia-Yin Chen, Xiao-Hui Wu, Yu-Ting Xue, Jiang-Bo Sun, Yong Wei, Qing-Shui Zheng, Xue-Yi Xue, Dong-Ning Chen, Ning Xu

**Affiliations:** 1 Department of Urology, Urology Research Institute, The First Affiliated Hospital, Fujian Medical University, Fuzhou 350005, China; 2 Department of Urology, National Regional Medical Center, Binhai Campus of the First Affiliated Hospital, Fujian Medical University, Fuzhou 350212, China; 3 Fujian Key Laboratory of Precision Medicine for Cancer, The First Affiliated Hospital, Fujian Medical University, Fuzhou 350005, China

**Keywords:** prostate cancer, liquid–liquid phase separation, biochemical recurrence, tumor immune microenvironment

## Abstract

To identify liquid–liquid phase separation (LLPS)-related molecular clusters, and to develop and validate a novel index based on LLPS for predicting the prognosis of prostate cancer (PCa) patients. We download the clinical and transcriptome data of PCa from TCGA and GEO database. The LLPS-related genes (LRGs) were extracted from PhaSepDB. Consensus clustering analysis was used to develop LLPS-related molecular subtypes for PCa. The LASSO cox regression analysis was performed to establish a novel LLPS-related index for predicting biochemical recurrence (BCR)-free survival (BCRFS). Preliminary experimental verification was performed. We initially identified a total of 102 differentially expressed LRGs for PCa. Three LLPS related molecular subtypes were identified. Moreover, we established a novel LLPS related signature for predicting BCRFS of PCa patients. Compared to low-risk patients in the training cohort, testing cohort and validating cohort, high-risk populations meant a higher risk of BCR and significantly poorer BCRFS. The area under receiver operating characteristic curve were 0.728, 0.762, and 0.741 at 1 year in the training cohort, testing cohort and validating cohort. Additionally, the subgroup analysis indicated that this index was especially suitable for PCa patients with age ≤ 65, T stage III-IV, N0 stage or in cluster 1. The *FUS*, which was the potential biomarker related to PCa liquid–liquid phase separation, was preliminarily identified and verified. This study successfully developed three LLPS-related molecular subtypes and identified a novel LLPS related molecular signature, which performed well in predicting BCRFS of PCa.

## 1. Introduction

Prostate cancer (PCa) is considered to be one of the most common malignant carcinomas of the male urinary system worldwide, and the incidence rate is increasing globally [1]. In a number of the latest updating guidelines and studies, radical prostatectomy (RP) and radical radiotherapy (RT) have been regarded as the curative therapy options for localized PCa [2,3]. Androgen deprivation therapy (ADT) is helpful to improve the outcome of high-risk PCa patients [4]. Additionally, 5α-reductase inhibitors (5-ARIs) block the conversion of testosterone into its active form, dihydrotestosterone (DHT), so that they are regarded as standard treatment strategies of benign prostate hyperplasia. Furthermore, they are related to the decrease in the incidence rate of PCa; however, the research results about their interaction with prostate cancer mortality (PCM) are inconsistent [5,6]. Then, biochemical recurrence (BCR) is a widely accepted indicator, indicating the poor prognosis of patients with PCa after treatments [7]. However, although many therapies have been proven to be beneficial for PCa patients, there are still a large proportion of PCa patients developing BCR after initial treatment. Therefore, almost all clinicians would focus on the serum prostate special antigen (PSA) level of PCa patients during treatment [8]. Sincerely, the identification of early BCR is helpful to the adjustment of patients’ treatment strategies. However, there were few recognized targets that could benefit the prediction of the BCRFS of PCa patients [9]. In brief, more effective predictors for the BCRFS and a deeper understanding of underlying mechanism have become an urgent need for clinical practice.

The development of cancer is believed to be intimately related to gene mutation or transcription disorder. Although much progress has been made in the pathogenesis of cancer recently, we still do not comprehend the specific mechanism of cancer progression entirely [10,11]. Most notably, it has been reported that membrane-less organelles, such as the nucleolus, exhibit significant liquid-like feature through a typical physicochemical process named liquid–liquid phase separation (LLPS) [12,13]. Based on it, the membrane-less organelles can dynamically exchange components with surrounding structures to maintain a relatively stable intracellular environment. Moreover, the relevant studies continuously revealed the correlation of LLPS with tumorigenesis and tumor development [13,14]. Several studies had reported the importance of LLPS in PCa treatment and progression [15]. Widely recognized, the resistance to anti-androgen therapy of patients with PCa might due to the androgen receptor (AR) mutations or splice variants restoring AR signaling [16]. The latest report demonstrated that the full-length AR could induce the LLPS in the cellular model of PCa [17]. Above all, the LLPS behavior of AR showed transcriptional activity and even anti-androgen efficacy. Therefore, targeting LLPS-mediated transcription factor (TF) and the formation of LLPS on enhancers to reprogram treatment-resistant tumor cells for preventing the progression of PCa seemed feasible [16,18]. Another study hypothesized that polyQ-dependent LLPS could potentially provide new therapies against castration-resistant prostate cancer (CRPC) by leading to the inhibition of transcriptional activity of AR [19]. However, to our knowledge, the studies which focus on exploring the correlation between LLPS and PCa, especially the BCR of PCa, are still limited.

In this study, we initially screened the differentially expressed liquid–liquid phase separation-related genes (DELRGs), and then identified three LLPS-related molecular clusters for PCa. Moreover, using least absolute shrinkage and selection operator (LASSO) analyses, we successfully developed a novel LLPS-related index based on six LRGs (including *FUS*, *CBX2*, *TPX2*, *TAZ*, *USH1C*, and *AXIN1*) that performed well in predicting the BCR-free survival (BCRFS) of patients with PCa. Then, external verifications were further performed. Particularly, the potential correlation of this novel signature with molecular subtypes, tumor immune microenvironment, and drug sensitivity were explored. Ultimately, preliminary experimental verification was performed to validate the biology for *FUS* which was the potential biomarker of PCa.

## 2. Results

### 2.1. Identification of Differentially Expressed LRGs and Functional Enrichment

**Figure 1:**
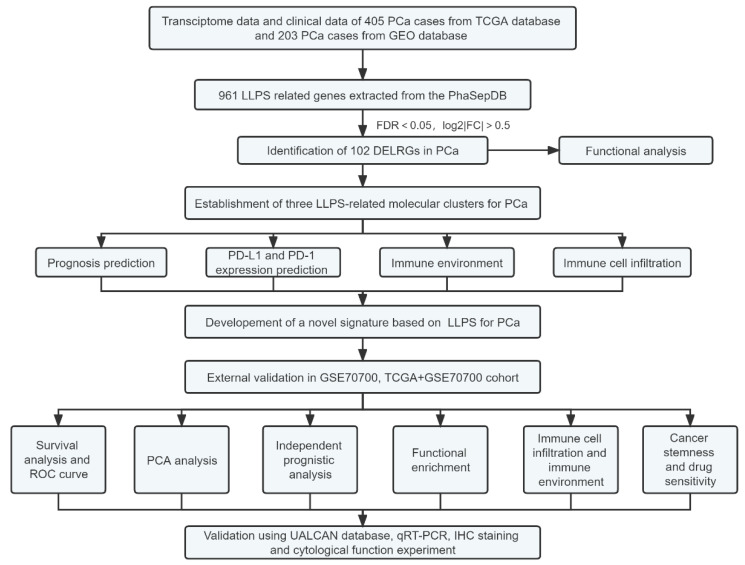
The flow diagram of our study.

The flow chart of this present study was demonstrated in Figure 1. We determined a total of 102 DELRGs between PCa and normal samples from TCGA database which were showed in Supplemental Table S4. Next, the heatmap and correlation network of these 102 DELRGs were shown in Supplemental Figure S1A,B, correspondingly. Moreover, the results of functional enrichment analysis of these 102 DELRGs were verified in Supplemental Figure S1C,D. In detail, the GO enrichment analysis demonstrated that the biological processes of these 102 DELRGs were significantly enriched in RNA splicing and regulation of mRNA metabolic process, while cellular component analysis showed these DELRGs were mainly enriched in ribonucleoprotein granule and nuclear speck. Respectively, the molecular function analysis verified that these 102 DELRGs were mainly enriched in mRNA 3’−UTR binding and modification-dependent protein blinding. Moreover, the KEGG pathway enrichment analysis indicated these 102 DELRGs were mostly enriched in amyotrophic lateral sclerosis, spliceosome, and cell cycle. In comparison to that in normal tissue, the expression of *PD-L1* in PCa tissue was markedly decreased (Supplemental Figure S1E). However, there was no significant diversity in the expression of *PD-1* between PCa and normal tissue (Supplemental Figure S1F).

### 2.2. Identification of Three Liquid–Liquid Phase Separation-Based Molecular Clusters

**Figure 2:**
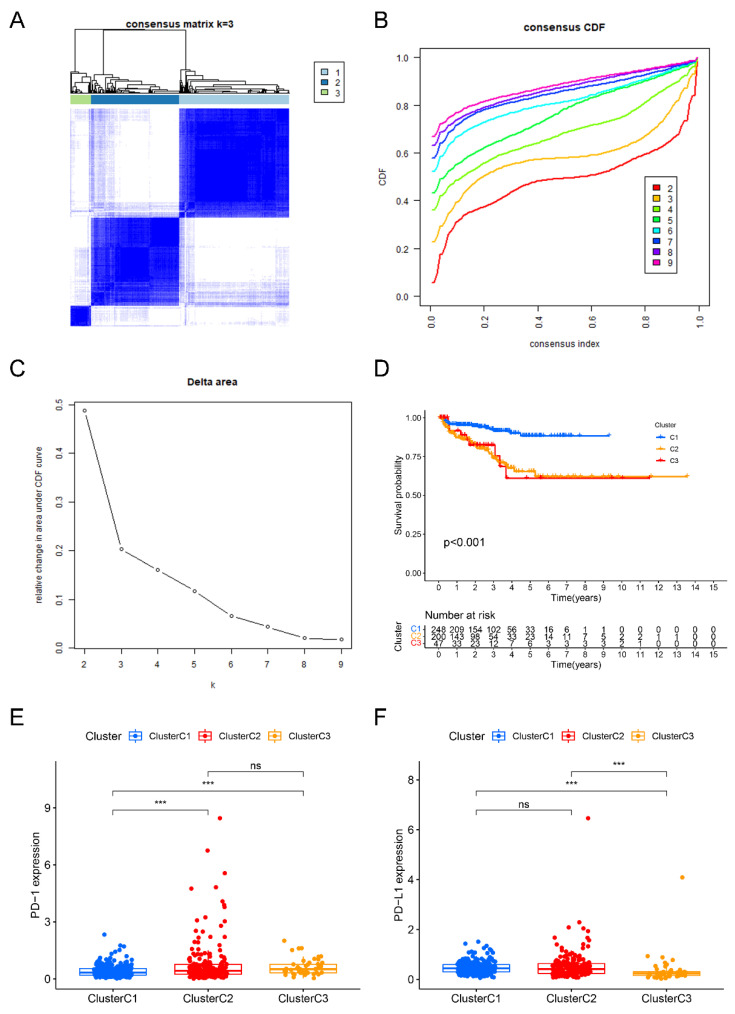
Consensus matrix heat map depicting consensus values on a white-to-blue color scale of each cluster (**A**). CDF plot displaying consensus distributions for each cluster (k) for PCa patients (**B**). Delta area plot reflecting the relative changes in the area under the CDF curve (**C**). The difference of biochemical recurrence free survival (**D**), the relative expression levels of *PD-1* (**E**) and *PD-L1* (**F**) among three clusters. ***: *p* < 0.001; ns: not significant.

Performing consensus clustering analysis, we successfully develop three liquid–liquid phase separation-based molecular clusters. In brief, the cluster 1 included 248 cases, cluster 2 included 200 cases, while the cluster 3 included 47 cases, respectively (Figure 2A–C). In addition, based on the results of Kaplan–Meier survival curves, we successfully verified that the difference of BCRFS among three LLPS-related clusters was significant (Figure 2D). Moreover, after comparing the relative expression levels of *PD-1* and *PD-L1* among these three LLPS-related clusters, we found that in comparison to that in cluster 1, the *PD-1* expression level in cluster 2 and cluster 3 was significantly increased; additionally, in comparison to that in cluster 3, the *PD-L1* expression level in cluster 1 and cluster 2 was markedly increased, respectively (Figure 2E,F).

**Figure 3:**
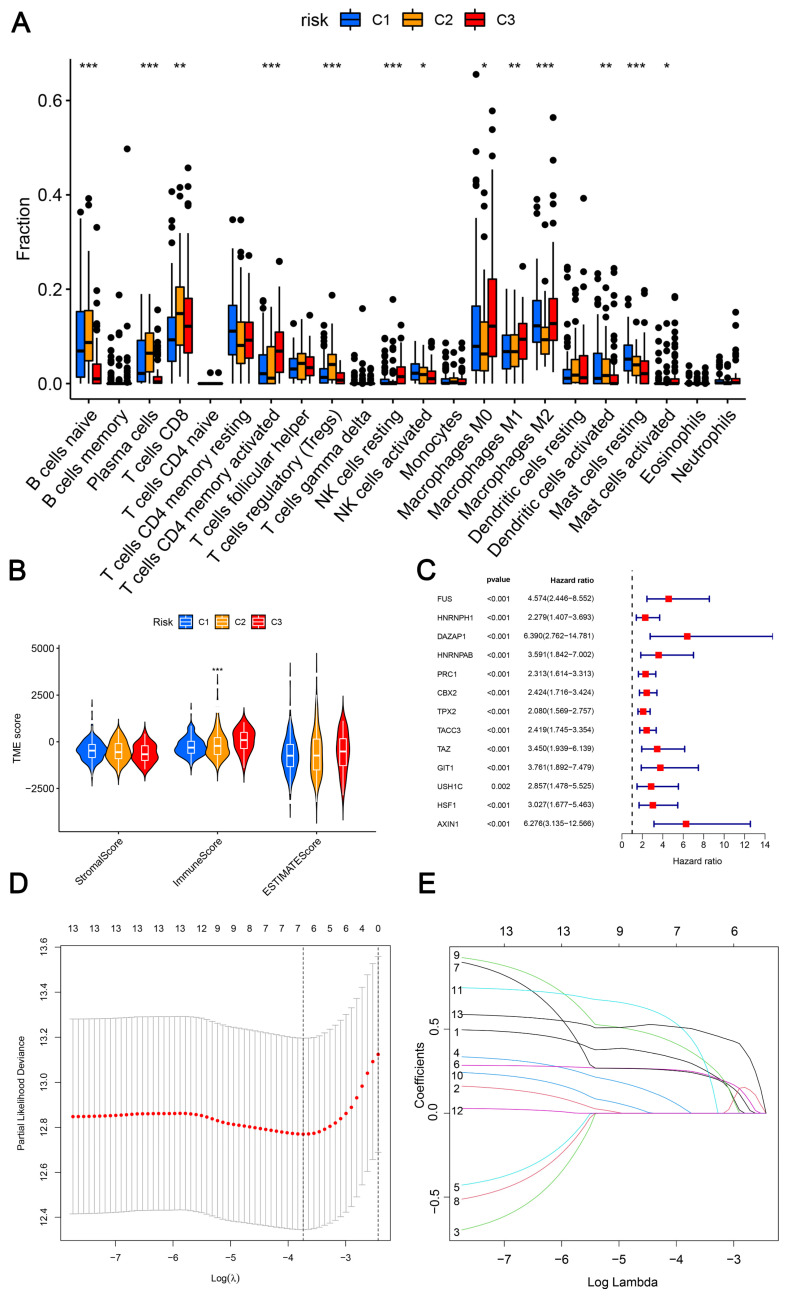
The correlation between three LLPS-related molecular clusters and immune cells infiltration (**A**), as well as tumor microenvironment scores (**B**). Identification of 13 prognostic LLPS-related genes based on univariate Cox regression analyses (**C**). Optimal predictor (lambda) selection in the lasso model: Lasso coefficients and vertical dashed lines were calculated at the best log (lambda) value, the red dots represented the target parameters corresponding to each lambda (**D**). The LASSO Cox analysis of the optimal prognostic DELRGs, the coefficients of the selected features are shown via the lambda parameter (**E**). *: *p* < 0.05; **: *p* < 0.01; ***: *p* < 0.001.

In the following study, naïve B cells, plasma cells, CD8^+^ T cells, activated memory CD4^+^ T cells, regulatory T cells (Tregs), activated NK cells, resting NK cells, M0 macrophages, M1 macrophages, M2 macrophages, activated dendritic cells, resting mast cells, and activated mast cells were the markedly different immune infiltrating cells among these three LLPS-related clusters (Figure 3A). Moreover, there were marked differences among these LLPS-related clusters in immune score, while no statistical difference was observed in the other TMB scores, including stromal score and ESTIMATE score (Figure 3B).

### 2.3. Development and Verification of a Novel Index Related to Liquid–Liquid Phase Separation for Predicting BCRFS

There was no remarkable difference in the basic data between training cohort and testing cohort (Table 1). Initially, through the training cohort, we successfully developed a novel index based on liquid–liquid phase separation. Notably, we screened a total of 13 DELRGs in the training cohort by performing univariable Cox regression analysis in the training cohort, which was based on the threshold *p*-value of 0.05 (Figure 3C). Next, focusing on these 13 DELRGs, we performed LASSO analysis to eventually screen 6 DELRGs (including *FUS*, C*BX2*, *TPX2*, *TAZ*, *USH1C*, and *AXIN1*) (Figure 3D,E). Finally, we constructed a novel prognostic index related to LLPS in order to benefit to predict BCRFS of PCa patients based on these six DELRGs. Most importantly, the calculation formula of risk score was as follows:

Risk score = (0.296152086400537) × *FUS* + (0.248773919618178) × *CBX2* + (0.241939073047545) × *TPX2* + (0.363669338326689) × *TAZ* + (0.4425683376976) × *USH1C* + (0.502952544015958) × *AXIN1*

According to median score of risk, we divided all cases into two subgroups (including high-risk and low-risk score subgroup). Moreover, we analyzed the risk score, the survival time and the expression heatmap of three cohorts, and the correlative results were demonstrated in Supplemental Figure S2.

**Figure 4:**
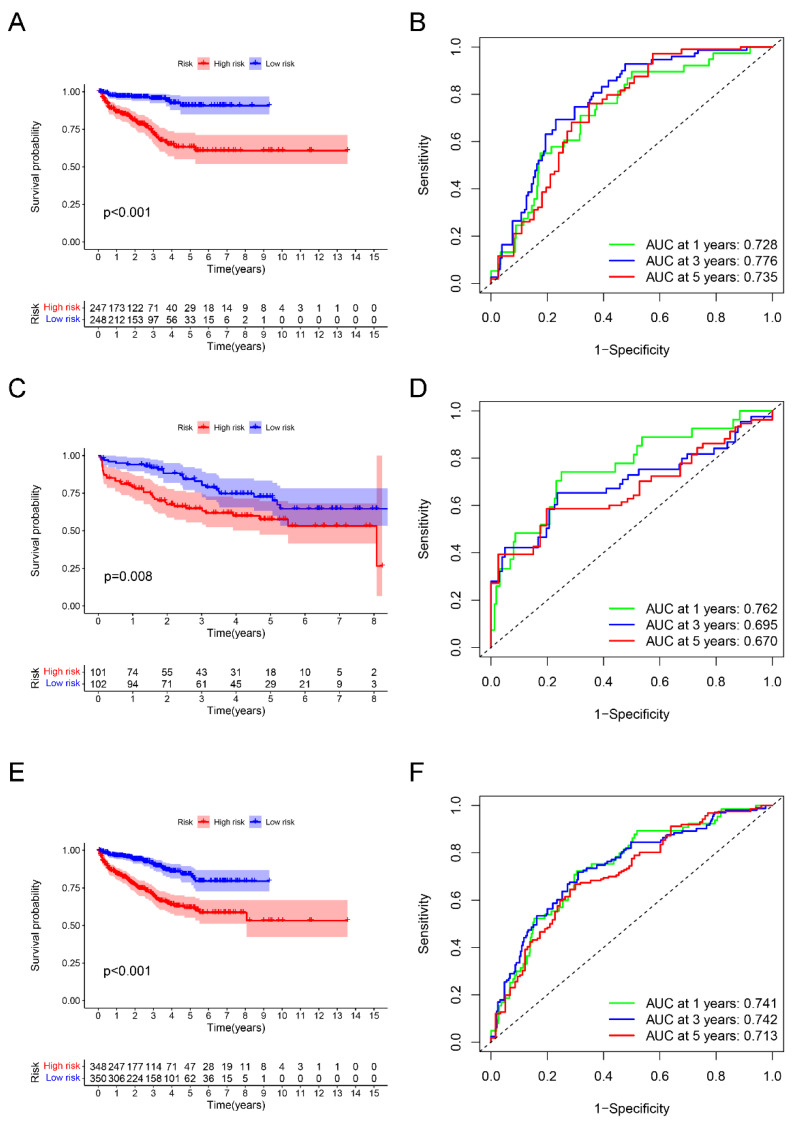
The survival analysis between high- and low-risk groups, and the corresponding area under ROC curve in the training cohort (**A**,**B**), testing cohort (**C**,**D**), and validating cohort (**E**,**F**).

Sincerely, between two subgroups, there were markedly significant differences of BCRFS among training cohort (*p* < 0.001), testing cohort (*p* = 0.008), and validating cohort (*p* < 0.001). Additionally, as those results of the area under time-dependent ROC curve showed, this novel index was helpful to predict the BCRFS of PCa cases (the area was 0.728, 0.776m and 0.735 at 1 year, 3 years, and 5 years in the training cohort; the area was 0.762, 0.695m and 0.670 at 1 year, 3 years, and 5 years in the testing cohort; the area was 0.741, 0.742, and 0.713 in the validating cohort) (Figure 4). Furthermore, the PCA analysis indicated that PCa patients with low- or high-risk scores were clearly located in two different distribution clusters (Supplemental Figure S3).

### 2.4. Subgroup Survival Analysis

**Figure 5:**
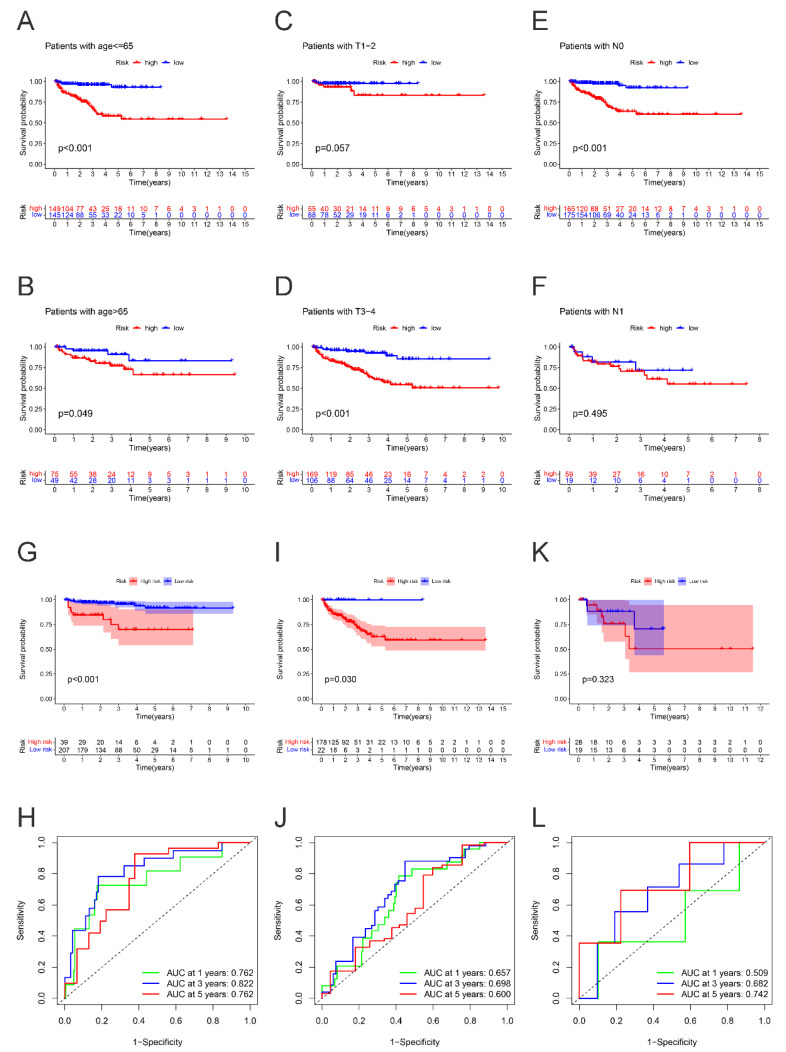
The subgroup survival analysis between high- and low-risk groups in PCa patients of age ≤ 65 (**A**), age > 65 (**B**), T stage 1–2 (**C**), T stage 3–4 (**D**), N0 stage (**E**), N1 stage (**F**). The survival analysis between high- and low-risk groups, and corresponding area under ROC curve in cluster 1 cohort (**G**,**H**), cluster 2 cohort (**I**,**J**), cluster 3 cohort (**K**,**L**).

Based on the results of subgroup survival analysis, we found that compared with patients with a low-risk score, high-risk PCa patients with age ≤ 65, T stage III-IV, or N0 stage might experience significantly worse BCRFS. Therefore, we indicated that this new LLPS-related index might be particularly suitable for PCa patients with age ≤ 65, T stage III-IV, or N0 stage. (Figure 5A–F).

Additionally, the following studies revealed that PCa patients with a high-risk score was related to significantly worse BCRFS in cluster 1 (*p* < 0.001) and cluster 2 (*p* = 0.030). Respectively, in cluster 3, we did not evaluate the marked difference of BCRFS between two subgroups in cluster 3 (*p* = 0.323). Additionally, the AUC for predicting BCRFS in cluster 1 was 0.762, 0.822, and 0.762 at 1 year, 3 years, and 5 years, respectively. The AUC for predicting BCRFS in cluster 2 was 0.657, 0.698, and 0.600, while in cluster 3 the AUC was 0.509, 0.682, and 0.742, respectively. The results further revealed that this novel index was especially suitable for PCa patients in cluster 1. (Figure 5G–L).

### 2.5. Clinicopathologic Characteristics, Caner Stemness Functional Enrichment, Cancer Stemness and Tumor Immune Microenvironment

**Figure 6:**
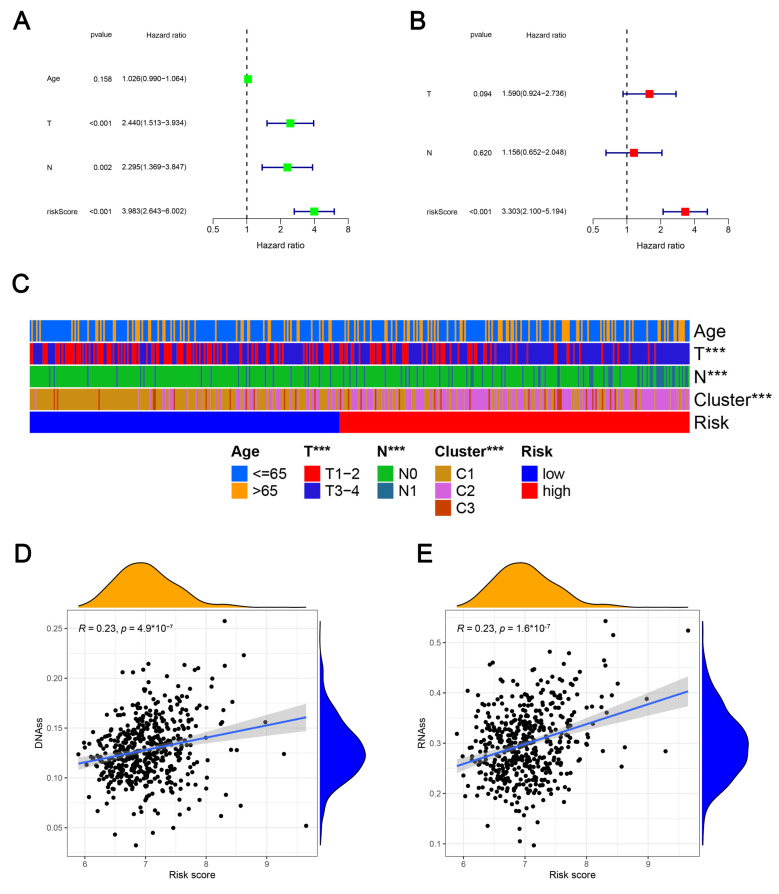
Univariate and multivariate independent prognostic analysis (**A**,**B**). Association of the LLPS-related signature with clinical characteristics (**C**), DNAss stemness score (**D**), RNAss stemness score (**E**). ***: *p* < 0.001.

Through independent analysis, we next explored that the risk score was the independent predictor for patients with PCa (Figure 6A,B). We also indicated the correlation between the risk score and some typical clinicopathologic features (Figure 6C), which demonstrated there were marked differences between high- and low-risk cases in T stage, N stage, and distinct clusters. The correlation analysis that followed demonstrated that different risk scores were positively associated with either DNA methylation-based stemness scores (DNAss) or mRNA expression-based stemness scores (RNAss) (Figure 6D,E). Using Gene Set Enrichment Analysis (GSEA) method, the Kyoto Encyclopedia of Genes and Genomes (KEGG) functional enrichment for both high-risk group and low-risk group was conducted. In low-risk PCa patients, the mainly functional enrichment were arrhythmogenic right ventricular cardiomyopathy, cardiac muscle contraction, dilated cardiomyopathy, hypertrophic cardiomyopathy, and tight junction, while in high-risk patients they were cell cycle, DNA replication, oocyte meiosis, ribosome, and spliceosome (Supplemental Figure S4A,B). Developing single-sample gene set enrichment analysis (ssGSEA) to quantify the immune functions, the results indicated that compared with low-risk patients, patients with high-risk had lower scores of mast cells, MHC class I, neutrophils Th1 cells, Treg, and Type II IFN response (Supplemental Figure S5A).

### 2.6. Anti-Cancer Drug Sensitivity Prediction

As demonstrated in the results of spearman correlation analysis, we merely showcased some desirable anti-cancer drugs. As the results showed, *FUS* performed well in predicting the sensitivity of Nelarabine (Cor = 0.448, *p* < 0.001), Hydroxyurea (Cor = 0.434, *p* < 0.001), and Pemetrexed (Cor = 0.392, *p* = 0.002). *USH1C* could be as the well-tried predictor of the sensitivity of Fluorouracil (Cor = 0.475, *p* < 0.001), Arsenic trioxide (Cor = −0.441, *p* < 0.001), and Everolimus (Cor = −0.376, *p* = 0.003). Moreover, *TAZ* could predict the drug sensitivity of Cladribine (Cor = 0.438, *p* < 0.001), Nelarabine (Cor = 0.424, *p* < 0.001), and Fludarabine (Cor = 0.404, *p* = 0.001). *TPX2* could also predict the drug sensitivity of Nelarabine (Cor = 0.380, *p* = 0.003). Correspondingly, *CBX2* was a hopeful predictor of the sensitivity of Dasatinib (Cor = −0.437, *p* < 0.001), Acrichine (Cor = 0.428 *p* < 0.001), and Nelarbine (Cor = 0.414, *p* < 0.001), while to Vinorelbine, *AXIN1* was also an expected predictor (Supplemental Figure S5B).

### 2.7. Validation mRNA Expression Levels of Risk Genes Using UALCAN Database

As demonstrated in UALCAN database, the relative mRNA expression levels of *FUS*, *CBX2*, *TPX2*, *TAZ*, and *AXIN1* were significantly more up-regulated in PCa tissues than in BPH tissues; there was not a remarkable difference in *USH1C* mRNA expression between PCa and BPH tissues, respectively (Supplemental Figure S6).

### 2.8. Verification of Relative mRNA Expression Levels of Risk DELRGs in RWPE-1 and PCa Cell Lines by Conducting qRT-PCR

**Figure 7:**
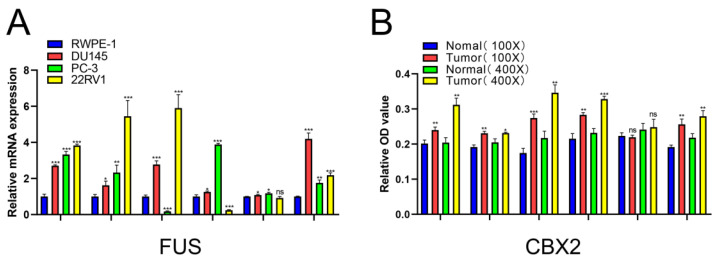

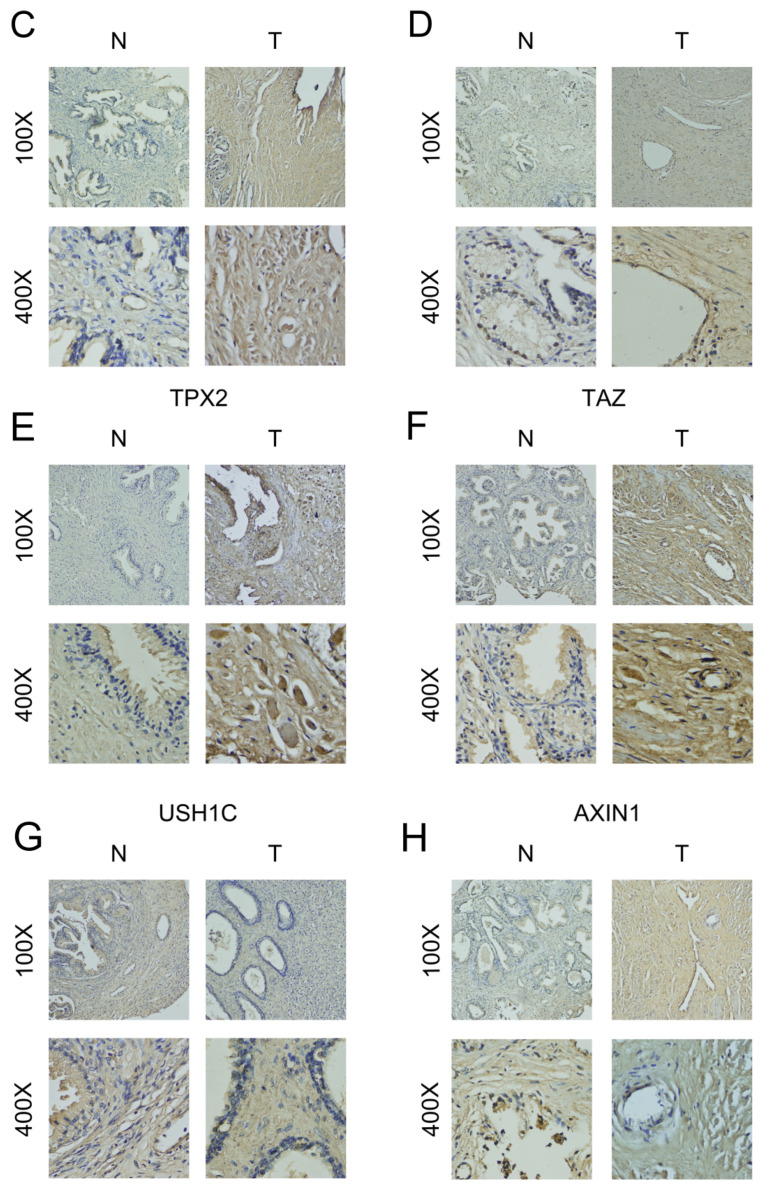
The mRNA expression levels between PCa cells and normal prostatic epithelial cells of *FUS, CBX2, TPX2, TAZ, USH1C* and *AXIN1* (**A**). The protein expression level between BPH and PCa tissue of FUS, CBX2, TPX2, TAZ, USH1C and AXIN1 (**B**–**H**). *: *p* < 0.05; **: *p* < 0.01; ***: *p* < 0.001; ns: not significant.

As mentioned above, this novel index was based on six DELRGs (including *FUS*, *CBX2*, *TPX2*, *TAZ*, *USH1C*, and *AXIN1*). Therefore, we next conducted qRT-PCR to evaluate mRNA expression levels of these target genes in RWPE-1, 22RV1, DU145, and PC-3 cell lines. Almost all experimental results were similar to the results of UALCAN database. The relative mRNA expression levels of *FUS*, *CBX2*, and *AXIN*1in all three PCa cell lines were significantly increased. More interestingly, those results revealed that compared with those in RWPE-1 cells, the relative mRNA expression levels of *TPX2* in 22RV1 and DU145 cell lines were remarkably up-regulated, while the relative mRNA expression level of *TPX2* in the PC-3 cell line was significantly down-regulated. To *TAZ* mRNA expression, it was markedly increased in DU145 and PC-3 cell lines, and was significantly decreased in 22RV1 cells when compared with that in RWPE-1 cells. Additionally, focusing on the mRNA expression level of *USH1C*, there were no marked expression differences in 22RV1 cells compared with that in RWPE-1 cells, which was consistent with the expected results. However, in both DU145 and PC-3 cell lines, it was markedly up-regulated (Figure 7A).

### 2.9. Verification of the Relative Protein Expression Levels of Six DELRGs in PCa Tissue through IHC Staining

Moreover, we developed IHC staining to indicate the difference of protein expression of these target genes between BPH and PCa tissues. It is worth mentioning that the BPH tissues were collected after transurethral resection of prostate and the tissue samples of PCa were collected after radical prostatectomy. Then, the results met the expected results, which successfully reconfirmed that the relative protein expression levels of FUS, CBX2, TPX2, TAZ, and AXIN1 were significantly increased in PCa tissues. Additionally, the difference of USH1C protein expression between PCa and BPH tissues was not significant (Figure 7B–H).

### 2.10. Inhibition of FUS Could Reduce Proliferation, Migration and Invasion, and Promote Apoptosis of PCa Cells

**Figure 8:**
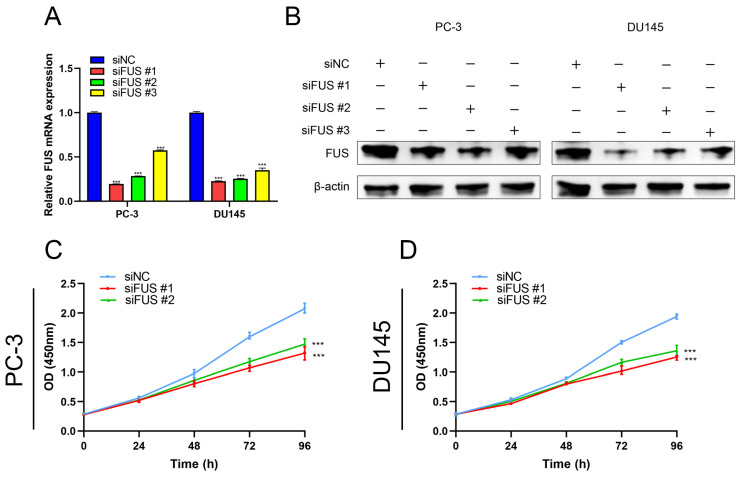

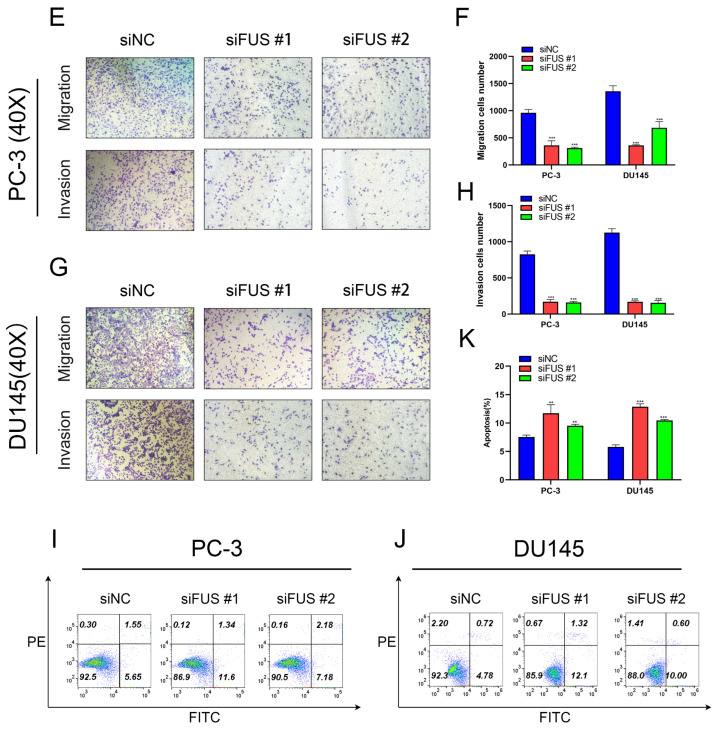
The biological function of *FUS* in the abilities of proliferation, migration, invasion, and apoptosis of PCa cells. The interference efficiencies of *FUS* in PC-3 and DU145 cells by siRNAs were verified by qRT-PCR (**A**) and Western blot (**B**). CCK-8 assays demonstrated the inhibition of *FUS* inhibit the proliferation ability of PC-3 and DU145 cells (**C**,**D**). Transwell assays indicated the depletion of *FUS* significantly blocked the migration and invasion ability of PC-3 and DU145 cells (**E**–**H**). The flow cytometric assay revealed that the inhibition of *FUS* could conspicuously promote apoptosis of PC-3 and DU145 cells (**I**–**K**). **: *p* < 0.01; ***: *p* < 0.001.

In recent studies, there has been limited focus on exploring the correlation between *FUS* and PCa development. So, we finally selected *FUS* for further verification, and chose DU145 and PC-3 cells for the next experiment. Initially, the results of qRT-PCR and Western blot were used for selecting two interfered targets with the highest interference efficiencies for further experiment (Figure 8A,B). CCK-8 assay verified that inhibition of *FUS* could reduce proliferation of PCa cells (Figure 8C,D). Transwell assay evaluated that the depletion of *FUS* could significantly alleviate migration and invasion of PCa cells (Figure 8E–H). Furthermore, the flow cytometric assay revealed that the inhibition of *FUS* could conspicuously promote apoptosis of PCa cells (Figure 8I–K).

## 3. Discussion

Previous studies have continually demonstrated that the development of cancer is closely related to genetic abnormalities, and that phase separation can drive tumor development [20]. The liquid–liquid phase separation (LLPS) especially acts on the dysregulation of epigenetics, which could lead to the tumorigenesis and tumor progression [21,22,23]. A number of recent studies consider LLPS as a novel therapeutic target of cancer intervention [21], similarly in prostate cancer [19]. However, there were limited studies that focused on exploring the correlation between LLPS and PCa, especially the BCR of PCa. In this present study, we primarily found DELRGs by using TCGA database. Most interestingly, based on the results of GO enrichment analysis, RNA-splicing and regulation of mRNA metabolic process was a key function among these genes. It is widely recognized that nearly all PCa patients would develop therapeutic resistance via diverse mechanisms including expression of AR splice variants (AR-Vs) [24]. Furthermore, we then found that among these DELRGs, *SFPQ* and *NONO* were included. *SFPQ* was proved to stabilize and activate AR-regulated gene expression, while knockdown of *NONO* was reported to inhibit the expression of AR-V7 more effectively than full-length *AR*. Moreover, the cooperative function of *SFPQ* and *NONO* could increase the expressions of genes associated with prostate cancer such as *AR* [25]. Then, the LLPS behavior of *AR* showed transcriptional activity and even anti-androgen efficacy. Targeting LLPS-mediated transcription factor (TF) to reprogram treatment-resistant tumor cells for preventing the progression of PCa was feasible. Therefore, we developed a total of three molecular clusters from the perspective of liquid–liquid phase separation, which was not yet reported.

As mentioned above, the identification of early BCR is helpful to adjust the treatment strategy of PCa patients. The sensitivity and specificity of PSA is limited. Therefore, new serum and urinary biomarkers have been developed in recent years. Nowadays, the latest advances of metabolomics make new potential biomarkers possible. Metabolomics plays a vital role for the progression and prognosis of PCa, and has been used for searching novel biomarkers of PCa recurrence. Particularly, choline phosphate has been regarded as a major biomarker for predicting the recurrence of PCa. Additionally, the exosomes are also a promising source of PCa biomarkers, which could also detected in plasma and urine [26]. As demonstrated in a prospective clinical trial, compared with standard serum PSA, the determination of PSA inside plasmatic exosomes shows higher performance in distinguishing BPH and PCa [27]. Although some studies have proposed potential biomarkers of BCRFS, there are still few recognized targets to help predict BCRFS in PCa patients. In this present study, using LASSO analyses, we succeeded in developing a novel prognostic index for predicting the BCRFS of PCa patients utilizing six DELRGs (including *FUS*, *CBX2*, *TPX2*, *TAZ*, *USH1C*, and *AXIN1*). A series of verification were then performed. The relative results indicated that PCa patients with a high-risk score were associated with significantly worse BCRFS compared to patients with low-risk scores in all three cohorts (including the training cohort, testing cohort, and validating cohort). Additionally, the area under time-dependent ROC curve was 0.728, 0.762, and 0.741 at 1 year in three cohorts sequentially, which could strongly demonstrate this novel signature performed well in predicting the BCRFS of PCa patients. Using univariate and multivariate independent prognostic analysis, the results emphatically indicated that this novel LLPS-related prognostic index was an independent predictor for BCR of PCa which showed a good application prospect.

The novel LLPS-related index was composed of six DELRGs: FUS RNA-binding protein (*FUS*), chromobox 2 (*CBX2*), TPX2 microtubule nucleation factor (*TPX2*), Tafazzin, phospholipid-lysophospholipid transacylase (*TAZ*), USH1 protein network component harmonin (*USH1C*), and *AXIN1*. All these six DELRGs have been revealed to participate in the tumorigenesis and tumor progression. He et al. [28] suggested that *FUS/circ_002136/miR-138-5p/SOX13* feedback loop played an irreplaceable role in regulating angiogenesis in glioma. Additionally, Chen et al. [29] indicated that *circHIF1A/NFIB/FUS* positive feedback loop played a critical role in the progression of triple-negative breast cancer (TNBC). *CBX2* had been proved to be highly expressed in Gastric cancer (GC) cells. Moreover, it could contribute to inhibiting the migration, proliferation, and invasion of GC cell lines via the *YAP/β-catenin* pathway, which indicated *CBX2* may represent a new therapeutic target of GC [30]. A number of studies reported the expression level of *TPX2* was related to poor prognosis of tumors [31,32]. Furthermore, Zhang et al. indicated that knockdown of *TPX2* repressed the tumor epithelial-mesenchymal transition (EMT) to inhibit tumor growth via *ERK/GSK3β/SNAIL* signaling pathway in prostate cancer [33]. Then, Li et al. reported that *miR-125a-5p* could negatively regulate the expression of *TAZ*, so that it could reverse the epithelial-mesenchymal transition (EMT) and restore the drug sensitivity in breast cancer [34]. Studies on the relationship between *USH1C* and tumor development were limited. Developing next-generation sequencing, Chen et al. found *USH1C* expression was down-regulated in 786-O cells after long-term hypoxia, and significantly bound up with the prognosis of patients with renal cancer [35]. As for *AXIN1*, Li et al. revealed *YTHDF2* participated in the progression of lung adenocarcinoma (LUAD) through upregulating he *AXIN1/Wnt/β-catenin* signaling, which might provide a new treatment strategy of LUAD [36]. Moreover, Zhang et al. also demonstrated *UBE3C* could activate the β-catenin signaling and further promote the development of GC via degrading *AXIN1* [37]. However, although the existing literature is large, whether there is a correlation between these six DELRGs and the BCRFS of PCa patients is still unclear. In this present study, we successfully developed a novel index based on six DELRGs (including *FUS, CBX2, TPX2, TAZ, USH1C*, and *AXIN1*), and revealed that these six DELRGs might be related to the BCR of PCa patients for the first time. Additionally, we ulteriorly validated mRNA and protein expression levels, which were approximately consistent with the expected results. Furthermore, recently, there had been limited studies contributing to exploring the correlation between *FUS* and PCa development, so we selected *FUS* for further preliminary experimental verification. The results successfully demonstrated that *FUS* played a regulator of proliferation, migration, invasion, and apoptosis of PCa cells. More importantly, all these results demonstrated the potential possibility of these six risk genes related to LLPS acting as the prognostic predictors of PCa. More research evidence was still needed.

It is widely reported that PCa has always been regarded as a cold tumor representing limited infiltration of immune cells and inadequate immune-related treatment response [38,39]. Recently, with some critical discoveries about advanced diagnostic platforms and immune mechanism, immunotherapy demonstrated the feasibility of treating PCa again, especially the castration-resistant prostate cancer (CRPC) [40]. A deeper understanding of the tumor immune microenvironment was urgently needed to improve the immunotherapy of PCa [38]. However, to our knowledge, there was still no clear conclusion about which types of PCa patients were more suitable for receiving immunotherapy. Many studies had indicated the *PD-L1* expression and phase separation might relate to the efficiency of immunotherapy [41,42]. In this study, we initially establish a total of three liquid–liquid phase separation-related molecular clusters of PCa, which exhibit significantly different *PD-1* or *PD-L1* expression levels, immune cell infiltrations, and immune environment. The high *PD-L1* expression was closely related to the poor clinical outcome of PCa patients [43]. A number of molecules could downregulate the *PD-L1* expression to inhibit PCa progression. Moreover, the interaction of *PD-1* with *PD-L1* was associated with immune escape. Both the expression of *PD-L1* and immune cell infiltration was required for improving the immunotherapy [39]. Moreover, based on this novel index, we then divided PCa cases into high- and low-risk subgroups. The correlation analysis demonstrated that different risk subgroups were positively associated with either DNAss cancer stemness score or RNAss cancer stemness score. Furthermore, the results of ssGSEA further revealed two subgroups also had significantly different tumor immune microenvironment. Therefore, this study may contribute to evaluating whether PCa cases benefit from immunotherapy, and even further improve the application of immunotherapy in prostate cancer. Such research is extremely meaningful.

However, several limitations are still needed to mention. First of all, this retrospective study was based on the bioinformatics method. Consequently, we still need the expected real-world data to further validate the feasibility this novel LLPS-related signature. Additionally, although several preliminary experiments were developed to validate the function of *FUS* in PCa development, further experiments in vitro and in vivo regarding the underlying mechanism of these six LLPS-related genes with PCa progression are still urgently required.

## 4. Materials and Methods

### 4.1. Data Collection and Preprocessing

First of all, this study was approved by the Institutional Ethics Committee of First Affiliated Hospital of Fujian Medical University and conducted in accordance with 1964 Helsinki declaration and its later amendments. All selected patients provided written informed consent before the sample collection.

We then downloaded the transcriptome data of 499 PCa samples and 52 normal samples from the Cancer Genome Atlas (TCGA) database (https://portal.gdc.cancer.gov/, accessed on 2 March 2022). After filtering, 405 PCa cases were included in this study, which were with complete data of transcriptome, BCR status, and BCR-free time. It deserves to be mentioned that only 70 samples have the data type of “days_to_first_biochemical_recurrence”, which was for the follow-up analysis. Additionally, GSE70770 (including 203 PCa cases with complete data of transcriptome, BCR status, and BCR-free time) was downloaded from Gene Expression Omnibus (GEO) databases (http://www.ncbi.nlm.nih.gov/geo/, accessed on 2 March 2022). Then, a total of 961 liquid–liquid phase separation-related genes were downloaded from PhaSepDB (http://db.phasep.pro/, accessed on 2 March 2022) [44] (Supplemental Table S1).

### 4.2. Identification of Differentially Expressed LRGs (DELRGs) and Functional Enrichment

R package “limma” and Wilcoxon test were used to identify LLPS-related genes (LRGs) between PCa cases and normal cases in TCGA datasets. Importantly, the cut-off value was set as *FDR* (false discovery rate) < 0.05 and log2 |fold change (*FC*)| > 1 [45]. Then, a Venn diagram was used to filter differentially expressed RRGs (DERRGs) by taking the intersection of DEGs and RRGs.

After conducting R packages “clusterProfiler”, “org.Hs.eg.db”, “enrichplot”, and “ggplot2”, the biological functions of the DELRGs were systematically explored by Gene Oncology (GO) and Kyoto Encyclopedia of Genes and Genomes (KEGG) analyses.

### 4.3. Establishment of LLPS-Related Molecular Clusters by Consensus Clustering Analysis

Initially, we conducted univariable Cox regression analysis to screen prognostic DELRGs associated with BCR-free survival (BCRFS) in whole TCGA cohort. The cut-off *p*-value was setting as 0.05. Next, R package “ConsensusClusterPlus” was utilized to identify LLPS-related molecular clusters. Furthermore, R packages “survival”, “survminer” and “pheatmap” were used to explore the relationship between LLPS-related subtypes with clinicopathologic characteristics. More importantly, the correlations of LLPS-related clusters with PD-L1 and PD-1 genes expression level was also explored. Respectively, using ESTIMATE method [46] and CIBERSORT algorithm [47], the dissimilarity of immune microenvironment and immune infiltrating cells among different LLPS-related molecular clusters were particularly revealed.

### 4.4. Establishment and Verification of a Novel LLPS-Related Prognostic Index for Prostate Cancer

In this study, we set the TCGA cohort (405 PCa samples) as training cohort. Respectively, the GSE70770 cohort (203 PCa samples) was set as the testing cohort. Additionally, a combined TCGA cohort and GSE70770 cohort was utilized as the validating cohort.

Initially, we conducted univariate Cox regression analysis and LASSO analysis to establish a novel LLPS-based molecular signature for predicting BCRFS based on package “glmnet”.

Moreover, using R package “Survminer”, we succeeded in calculating the optimal cutoff value (OCV). On the basis of the OCV, we next divided all PCa patients into high- and low-risk subgroups. Furthermore, based on R packages “survival”, “survminer”, ”timeROC”, and “Rtsne”, survival analysis, time-dependent on the receiver operating characteristic (ROC) curve analysis and principal component analysis (PCA), was utilized to validate the performance of this novel LLPS-based index. Most importantly, we ulteriorly performed external validation in testing cohort and validating cohort, respectively.

Likewise, we also performed independent prognostic analysis to demonstrate whether the index could predict the BCRFS in PCa independently of other clinicopathologic features. According to R package “ComplexHeatmap”, we successfully presented the relevance between prognostic target and other clinicopathologic features. Ultimately, through packages “survival”, “survminer” in R, we performed survival analysis in multiple subgroups including age, grade, and AJCC-stage.

### 4.5. Association of the LLPS-Related Signature with Clinicopathologic Features, Tumor Stemness Scores, Immune Microenvironment, and Functional Enrichment

Then, we testified the relationship of this novel LLPS-related signature with the relative expression levels of PD-L1, clinicopathologic features, tumor immune microenvironment, immune cells infiltration, immune-related pathways activity, and tumor stemness scores. In detail, tumor immune microenvironment scores were assessed by using ESTIMATE algorithm while 13 immune function activities were estimated by performing Single-sample gene set enrichment analysis (ssGSEA). Moreover, gene set enrichment analysis (GSEA) was developed to investigate functional enrichment related to this LLPS-based index [48].

### 4.6. Drug Sensitivity of Risk DELRGs

Using CellMiner database (https://discover.nci.nih.gov/cellminer/home.do, accessed on 4 March 2022), which is designed for cancer research, we successfully investigated whether the DELRGs could act as a predictor of the sensitivity of anti-cancer drugs by performing Spearman correlation analysis. The drugs included in the analysis were either FDA approved or undergoing clinical trials were to enhance the correlation between the LLPS-related genes and clinical application.

### 4.7. Validation of Risk Genes Using UALCAN

UALCAN database (http://ualcan.path.uab.edu/, accessed on 5 March 2022), was used for testifying the relative mRNA expression levels of risk DELRGs between normal prostate and PCa cancer tissues.

### 4.8. Cell Culture

RWPE-1, 22RV1, PC-3, and DU145 cell lines were used in subsequent validation experiments. All these cell lines were obtained from Procell Life Science and Technology Co., Ltd. (Wuhan, China). In brief, RPMI-1640 medium (Biological Industries, Beit Haemek, Israel) with 10% fetal bovine serum (FBS) was used to culture RWPE-1 cell line and 22RV1 cell line. Comparatively, the DU145 and PC-3 cells were cultured in corresponding cell specific medium. All these cell specific media were also provided by Procell Life Science and Technology Co., Ltd (Wuhan, China). Most importantly, the cells were strictly incubated at 37 degrees and 5% CO_2_.

### 4.9. Quantitative Reverse-Transcription Polymerase Chain Reaction (qRT-PCR)

The total RNA of cells were collected using FastPure Cell/Tissue Total RNA Isolation Kit V2 (RC112-01, Vazyme). The RNA was reverse-transcribed (HiScript III All-in-one RT SuperMix Perfect for qPCR, R333-01, Vazyme) and the risk DELRGs were detected with qPCR (Taq Pro Universal SYBR qPCR Master Mix, Q712-02, Vazyme) with the Step One PlusTM PCR System (Applied Biosystems, Waltham, MA, USA). The primer sequences were presented in Supplemental Table S2.

### 4.10. Immunohistochemical (IHC) Staining

Patients with benign prostatic hyperplasia (BPH) and patients with PCa were biopsiedin the Department of Urology of the First Affiliated Hospital of Fujian Medical University. After pathological diagnosis, they were treated eventually surgically. The tissue samples of BPH were collected after transurethral resection of prostate, while the tissue samples of PCa were collected after radical prostatectomy. In brief, these fresh tissues were embedded into wax blocks which were then processed into 5 μM thick sections. The eaduse immunohistochemical UltraSensitiveTM SP kit (KIT-9710) was obtained from Maixin biotechnologies. Based on the manufacturer’s protocol, after non-specific dye blocking, the sections were subsequently incubated with the anti-FUS (YT1800, Immunoway), anti-CBX2 (YT0698, Immunoway), anti-TPX2 (YT4712), anti-TAZ (YN1701, Immunoway), anti-USH1C (11358-1-AP, Proteintech) and anti-AXIN1 (YN0494. Immunoway) at 4 °C overnight. In the next day, biotin-labeled sheep anti-mouse/rabbit IgG polyme was used to cover the sections. The Phosphate Buffered Saline (PBS) solution was required to be rinsed three times between each step. Finally, the sections went through DAB staining, and then pictures were taken. The results were processed by the ImageJ software, and the average optical density (AOD) was used as an indicator of the relative expression [9].

### 4.11. RNA Interference

According to instructions, cells were transfected with small-interfering RNAs (siRNAs) using Lipofectamine 2000 reagent (Thermo Fisher Scientific, Waltham, MA, USA). The siRNAs were purchased from GenePharma Co., Ltd. (Shanghai, China). (Sequences were presented in Supplemental Table S3).

### 4.12. Western Blotting

In brief, the PCa cells were added into RIPA buffer (P0013B, Beyotime, Shanghai, China), and the BCA assay kit (P0010, Beyotime) were used for the determination of protein. The proteins were firstly separated in 10% SDS-PAGE, then transferred to PVDF membranes (Millipore, USA). The membranes were blocked in NcmBlot blocking buffer (P30500, NCM Biotech, Suzhou, China), and incubating the membranes at 4 °C overnight by covering specific primary antibodies. The primary antibodies contained anti-Actin (YM3028, Immunoway, 1/5000) and rabbit anti-FUS (YT1800, Immunoway, 1/1000). Then, the membranes were covered with HRP-conjugated anti-rabbit IgG (RS0002, Immunoway, 1/10000) for 1 h. ECL reagent (K-12045-D10, Advansta, Menlo Park, CA, USA) was used to detect the levels of protein.

### 4.13. CCK-8 Viability Assays

The CCK-8 assay kit (MA0218, Meilunbio, Dalian, China) was used for accessing the proliferation. The PCa cells were seeded onto 96-well plates with a density of 5000 cells per well. Then the cells were incubated for 24, 48, 72, or 96h. Next, the complete medium containing 10% CCK-8 assay kit was added into each well. The plates needed to incubated for about 1h. Finally, the absorbance was determined with a multiplate reader at 450 nm.

### 4.14. Transwell Migration and Invasion Assays

The transwell plates (24-well, 8.0-μm, Corning, Shanghai, China) were used to conduct the PCa cell migration and invasion experiment. The transfected PCa cells (3 × 10^4^) were resuspended using serum-free medium. Cell suspension was added into the upper inserts of Corning plates for both migration and invasion assays. Particularly, the inserts needed to precoat with 20 μg Matrigel (Corning, Shanghai, China) for invasion assays. Next, about 500 µL medium with 20% FBS was added into the lower inserts. Importantly, the plates were incubated for about 48 h in an incubator. Cells attached to the membrane were fixed via 4% paraformaldehyd for 30 min. Through 25 minutes’ dyeing by 0.1% crystal violet, membranes were observed through a microscope and results were processed via using ImageJ software.

### 4.15. Flow Cytometry

The Annexin V-FITC/PI detection kit (A211-01, Vazyme, Nanjing, China) was used to evaluate apoptosis. In brief, cell pellets were resuspended with 100 µL 1× binding buffer, incubated by 5 µL Annexin V-FITC and 5μL PI for about 10 min, and then added to 400 μL 1× binding buffer to mix again. NovoCyt^TM^ Flow cytometry (NovoCyte 2060R, ACEA BIO Co., Ltd., Hangzhou, China) was used for measuring and analysis.

### 4.16. Statistical Analysis

Each experiment was performed at least three times independently. The analyses were developed via SPSS 19.0 software (IBM, Armonk, NY, USA) and Prism version 8.0 (GraphPad Software, San Diego, CA, USA). Importantly, the data were presented as mean ± SD unless otherwise stated. Using the Student’s test to analyze the statistical significance between two independent groups. The *p* value of <0.05 was considered statistically significant.

## 5. Conclusions

To summarize, this study identified three LLPS-related molecular clusters with significantly distinct immune microenvironment and clinical characteristics. Moreover, we successfully established a novel LLPS-related index based on six DELRGs (including *FUS*, *CBX2*, *TPX2*, *TAZ*, *USH1C*, and *AXIN1*), which performed well in predicting the BCRFS and might be important to PCa development and progression. Preliminary experimental verification demonstrated that *FUS* could regulate the proliferation, migration, invasion, and apoptosis of PCa cells. All these findings would contribute to a deeper understanding of potential mechanism of phase separation affecting the progression and BCR of PCa patients.

## Figures and Tables

**Table 1:** The basis data of training cohort and testing cohort.

Variables	Training Cohort	Testing Cohort	*p*-Value
Age	60.87 ± 6.59	60.98 ± 6.86	0.869
T stage	—	—	0.349
T2a	1 (0.5%)	5 (2.5%)	—
T2b	4 (2.0%)	5 (2.5%)	—
T2c	62 (30.4%)	73 (36.3%)	—
T3a	69 (33.7%)	62 (30.8%)	—
T3b	62 (30.4%)	52 (25.9%)	—
T4	2 (1.0%)	3 (1.5%)	—
Unknown	4 (2.0%)	1 (0.5%)	—
N stage	—	—	0.252
N0	148 (72.6%)	142 (70.7%)	—
N1	34 (16.7%)	27 (13.4%)	—
Unknown	32 (15.7%)	32 (15.9%)	—

## Data Availability

All data generated or analyzed during the present study was downloaded from TCGA database (https://portal.gdc.cancer.gov, accessed on 2 March 2022), and GEO database (https://www.ncbi.nlm.nih.gov, accessed on 2 March 2022).

## Supplementary Materials

The following supporting information can be downloaded at: https://www.mdpi.com/article/10.3390/ijms24065515/s1.
