# Supplementary material for: A Liquid–Liquid Phase Separation-Related Index Associate with Biochemical Recurrence and Tumor Immune Environment of Prostate Cancer Patients
Source: Int J Mol Sci. 2023 Mar 14;24(6):5515. doi: 10.3390/ijms24065515 (PMC10058551; doi:10.3390/ijms24065515)
Supplement: Supplementary file 1 [file ijms-24-05515-s001.zip › Supplemental Figure S1-S6.pdf]

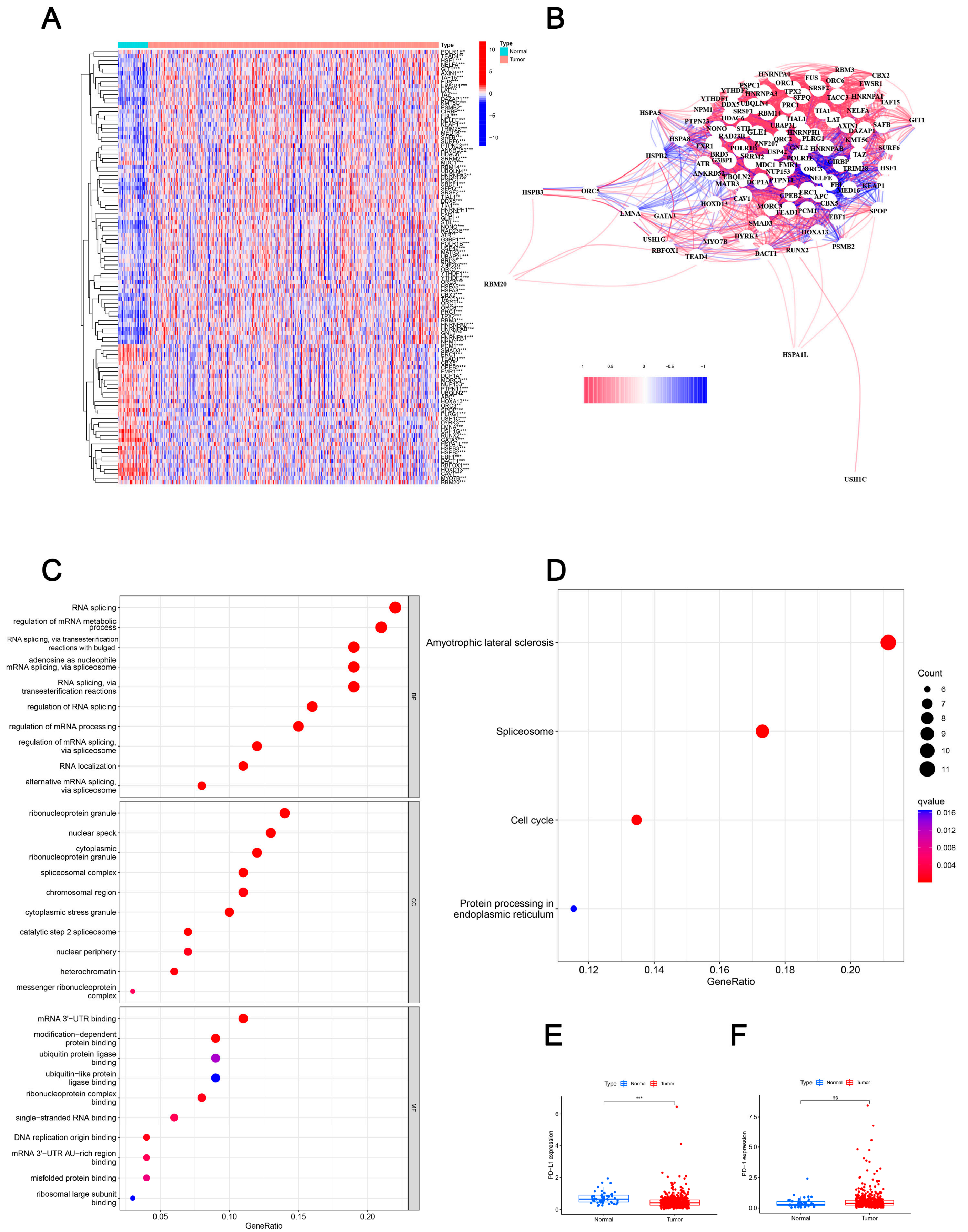

Figure S1. The heatmap of 102 DELRGs (**A**), the correlation network of 102 DELRGs (**B**), the GO enrichment analysis (**C**) and the KEGG pathway enrichment analysis (**D**) of 102 DELRGs, the PD-L1 expression (**E**) and PD-1 expression (**F**) between PCa tissues and normal tissues. ***: $p < 0.001$; ns: not significant.

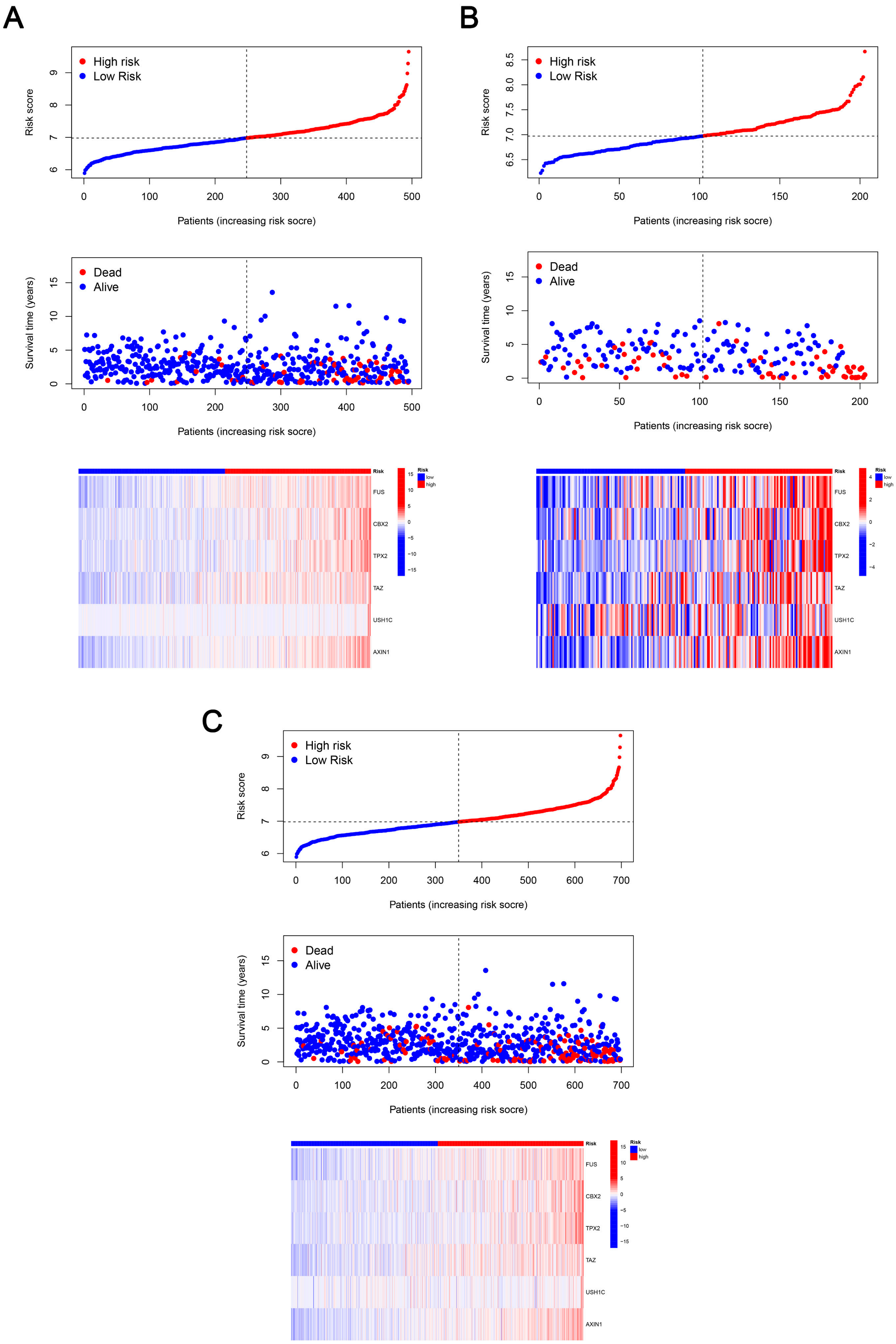

Figure S2. Development and validation of a novel LLPS-related prognostic index for PCa. The risk score, the survival time and the expression heatmap of training cohort (**A**), testing cohort (**B**) and validating cohort (**C**).

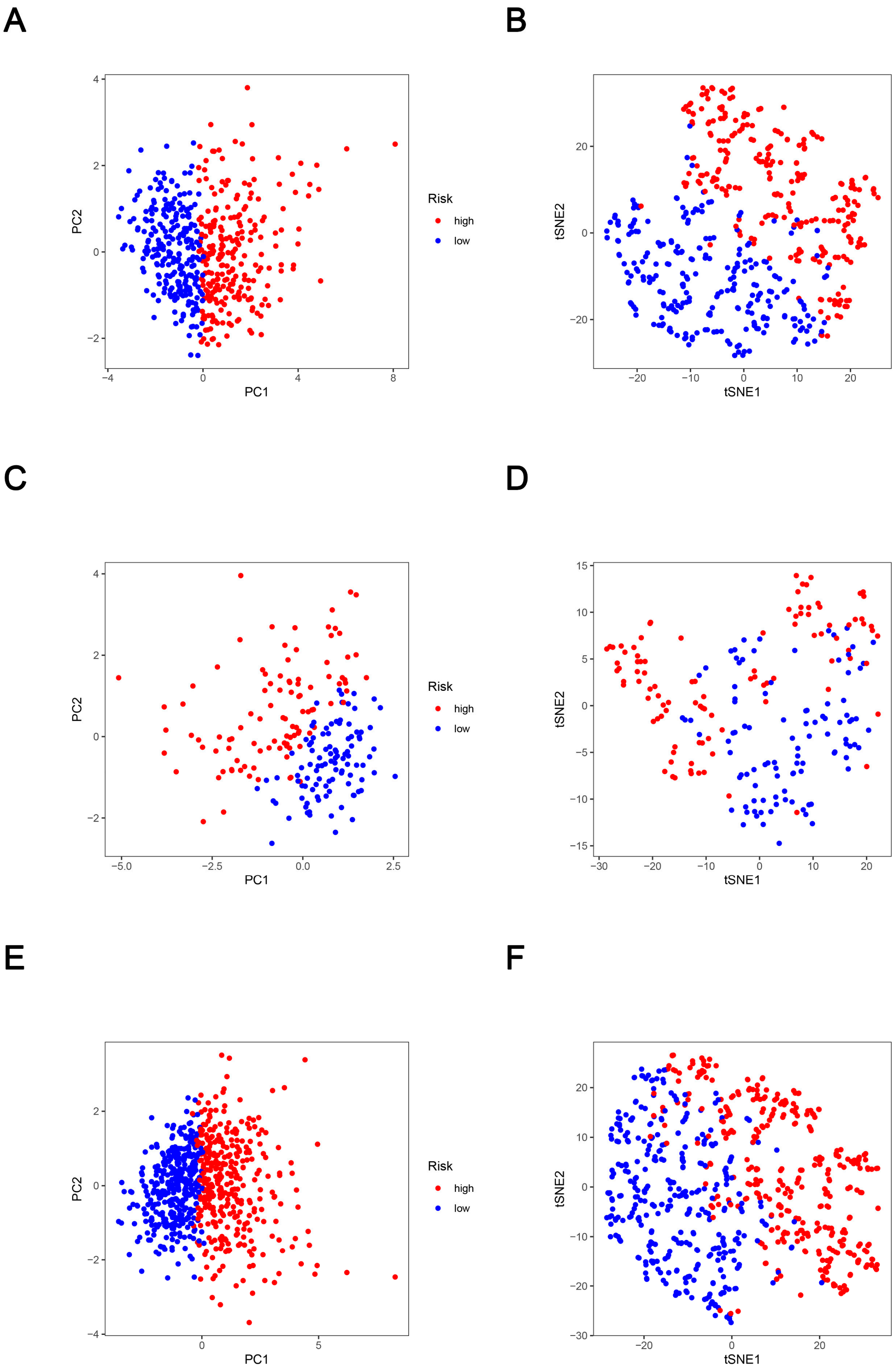

Figure S3. The PCA analysis of training cohort (**A**,**B**), testing cohort (**C**,**D**) and validating cohort (**E**,**F**).

A

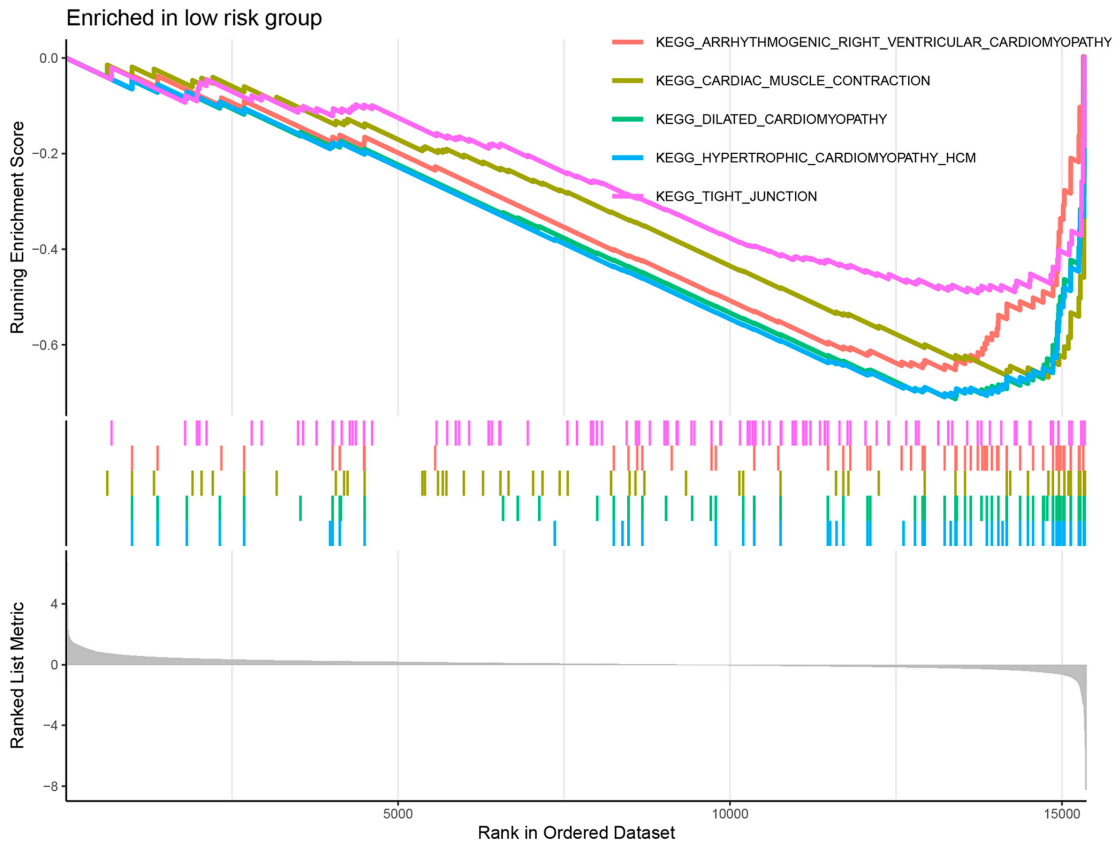

B

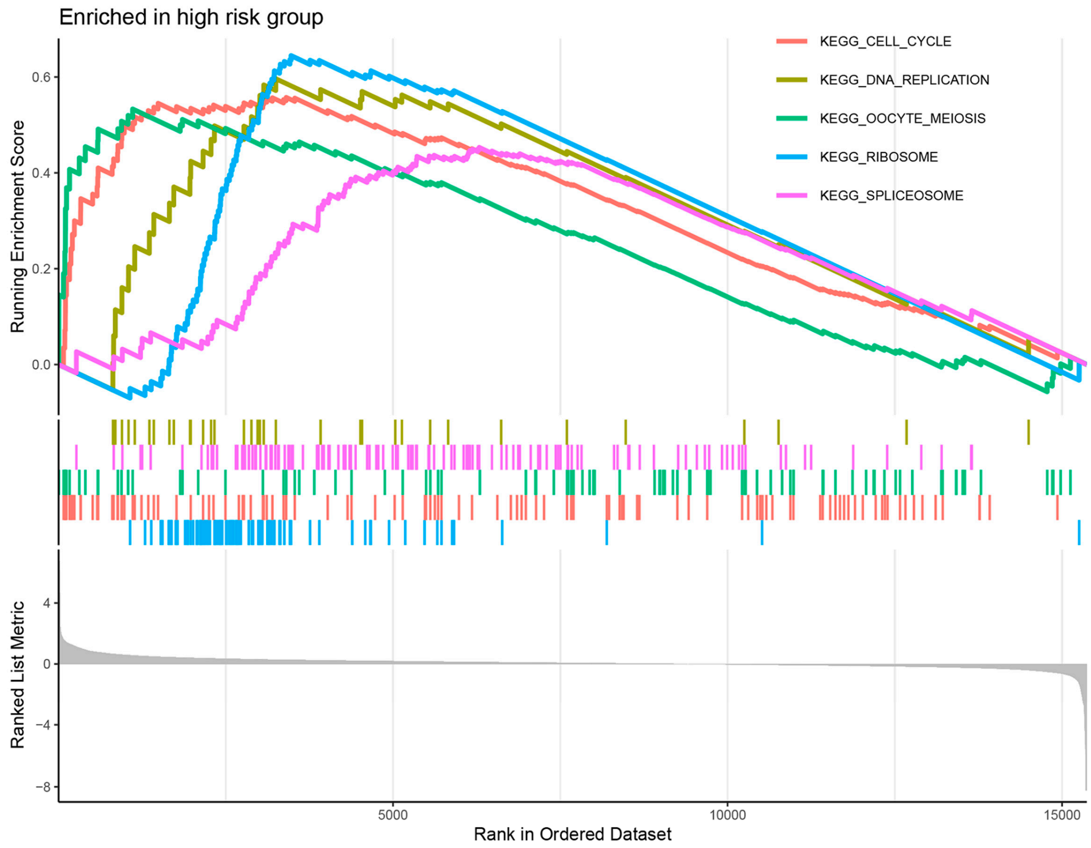

Figure S4. The KEGG functional enrichment for both low-risk group (**A**) and high-risk group (**B**).

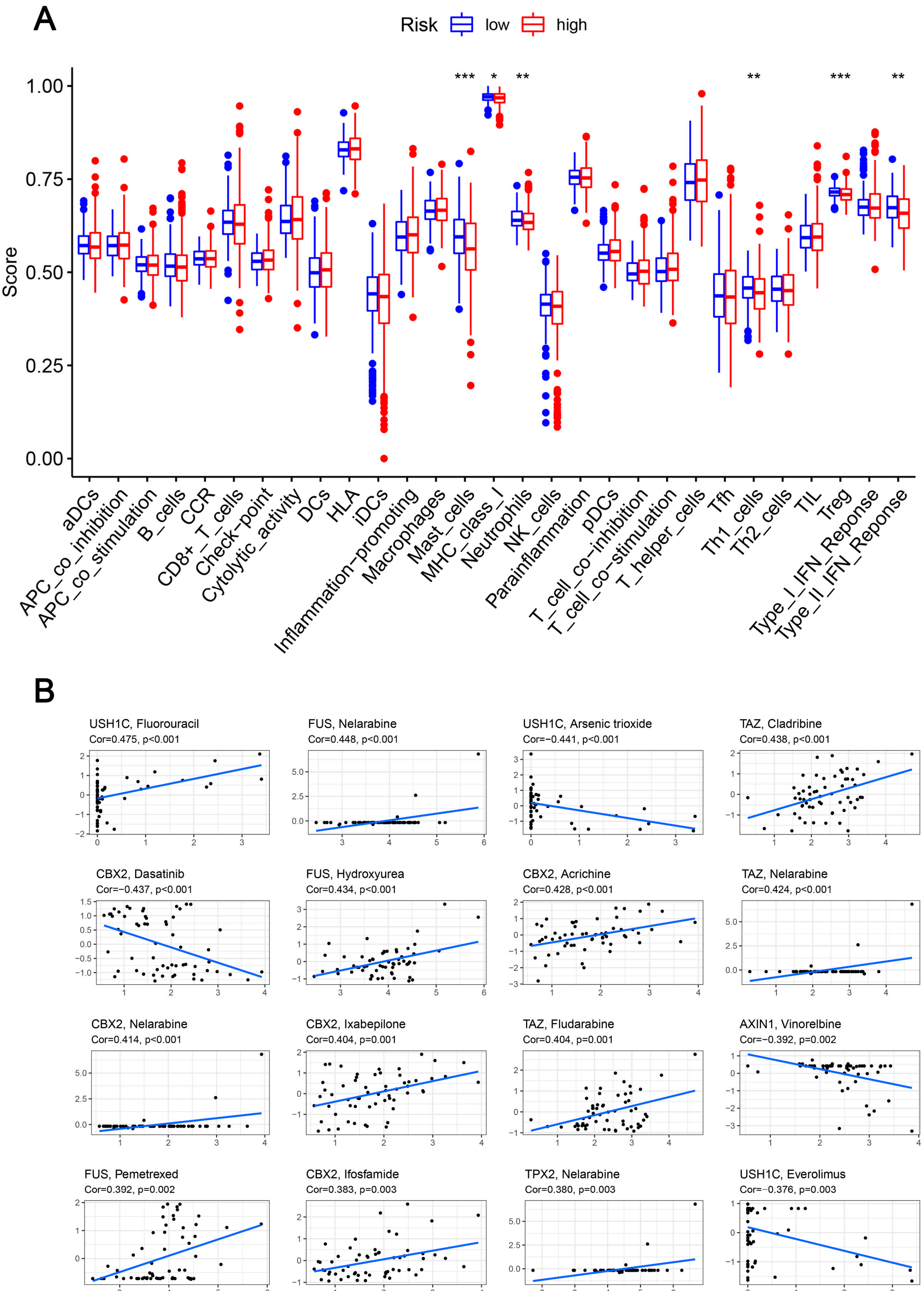

Figure S5. Association of this LLPS-related signature with tumor immune microen-vironment (**A**) and an-ti-cancer sensitivity (**B**). *: $p < 0.05$; **: $p < 0.01$; ***: $p < 0.001$.

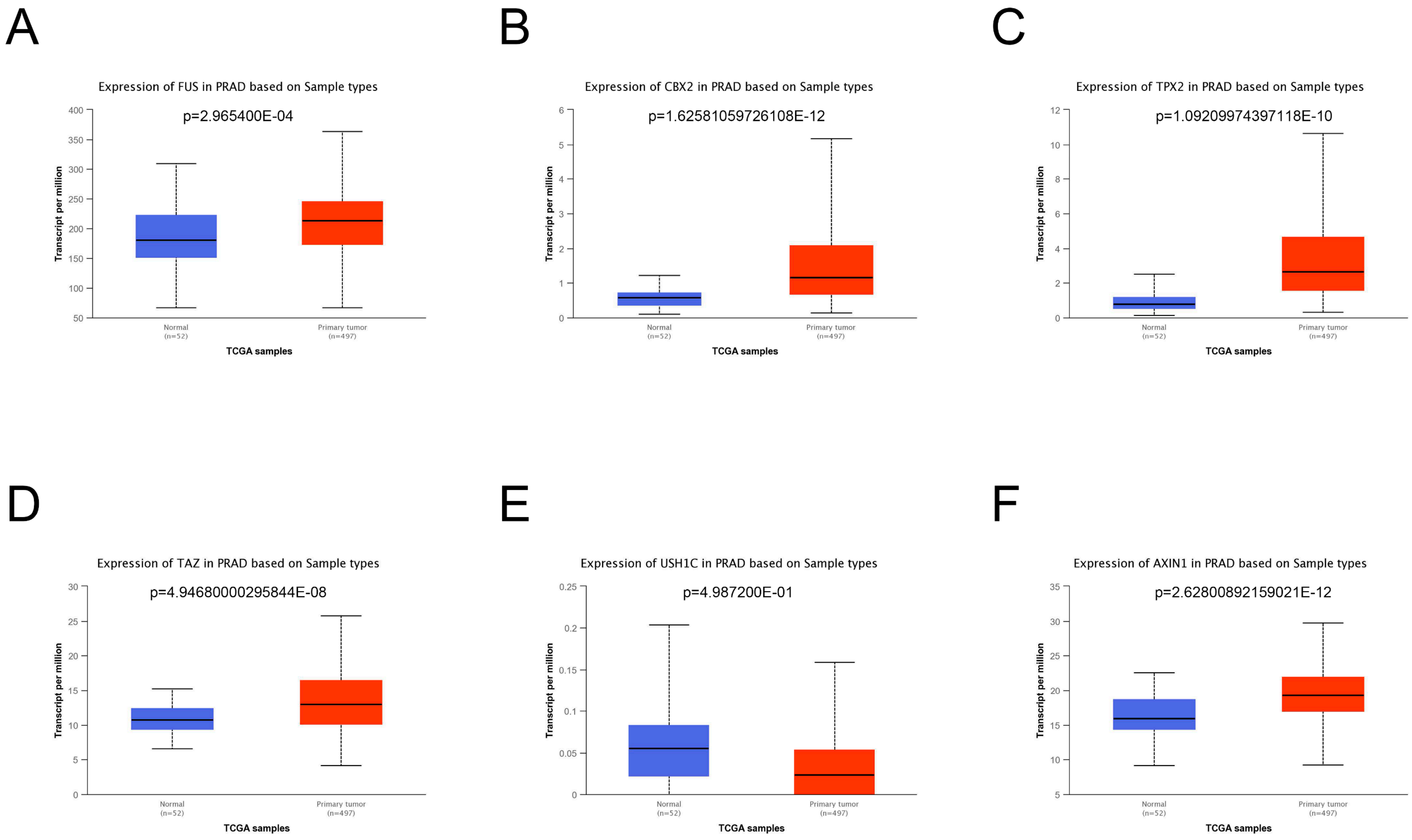

Figure S6. Validation mRNA expression levels of *FUS* (**A**), *CBX2* (**B**), *TPX2* (**C**), *TAZ* (**D**), *USH1C* (**E**) and *AXIN1* (**F**) using UALCAN database.
