# Supplementary material for: A Liquid–Liquid Phase Separation-Related Index Associate with Biochemical Recurrence and Tumor Immune Environment of Prostate Cancer Patients
Source: Int J Mol Sci. 2023 Mar 14;24(6):5515. doi: 10.3390/ijms24065515 (PMC10058551; doi:10.3390/ijms24065515)
Supplement: Supplementary file 1 [file ijms-24-05515-s001.zip › Supplemental Tables S2--S4.pdf]

Supplemental Table S2. Sequences of primers used for qRT-PCR.

Genes	Sequences
**AXIN1**	
F	5’-CTGGATACCTGCCGACCTTA-3’
R	5’-CCTTCGCTGTACCGTCTACT-3’
**CBX2**	
F	5’-GACTTAGATGCTAAGAGGGGTC-3’
R	5’-CTTCTTCCGGATGGGATCCTTC-3’
**FUS**	
F	5’-TGGTGACTGGAAGTGTCCTAAT-3’
R	5’-CCTCGATCATAGCCTCCTCTG-3’
**GAPDH**	
F	5’-GGTGTGAACCATGAGAAGTATGA-3’
R	5’-GAGTCCTTCCACGATACCAAAG-3’
**TAZ**	
F	5’-CAGCAATGTGGATGAGATGGAT-3’
R	5’-TGGAAGACAGTCAAGGAAATCAG-3’
**TPX2**	
F	5’-GCCTTTCTGGTTCTCTAGTTCAG-3’
R	5’-GGGCTTCTACCTCAGCCATT-3’
**USH1C**	
F	5’-ATGGCAATCAACGGCAAGAT-3’
R	5’-GCTCATCGTCATACTCCTTTGG-3’

Supplemental Table S3. Sequences

Product	Sense (5’-3’)	Antisense (5’-3’)
Control siRNA (si-NC)	UUCUCCGAACGUGUCACGUTT	ACGUGACACGUUCGGAGAATT
siFUS #1	CGUGGUGGCUUCAAUAAAUTT	AUUUAUUGAAGCCACCACGTT
siFUS #2	CAGCCCAUGAUUAAUUUGUTT	ACAAAUUAAUCAUGGGCUGTT
siFUS #3	CCACCUGUGAGAAUAUGAATT	UUCAUAUUCUCACAGGUGGTT

Supplemental Table S4. Identification of differentially expressed liquid-liquid phase separation related genes.

Gene	Normal Mean	Tumor Mean	Log2|FC|	*p* Value
NUP153	8.579793808	7.662285172	−0.163168256	0.013512463
TIA1	8.112758269	10.61429345	0.387743933	0.000130968
FUS	17.03956577	23.1901605	0.444624234	1.69E-10
RBM3	40.88157231	48.78208493	0.254900736	0.000439331
CIRBP	60.90626481	79.16102898	0.378199734	1.22E-08
CPEB2	4.110357558	3.431697038	−0.260341706	3.56E-05
TAF15	22.46660529	28.12779998	0.324214567	1.63E-11
RBM14	11.62654231	14.09037796	0.277288203	1.98E-10
RBFOX1	0.285413496	0.092323924	−1.628277114	8.67E-16
HNRNPA1	98.41739538	121.0395393	0.29849316	1.70E-08
UBQLN2	17.96799865	16.17065929	−0.152051225	0.001955176
EWSR1	19.79538692	23.29406104	0.234797911	3.91E-08
HNRNPH1	20.31015058	24.07233193	0.24517497	0.000581284
HNRNPA3	37.67330942	40.7208279	0.11222412	0.008933498
TIAL1	9.563817769	10.45385907	0.12837707	0.003976384
HNRNPA0	19.47344077	22.81610454	0.22854468	8.56E-06
PSPC1	9.642304615	11.8932142	0.302688751	2.27E-09
DAZAP1	7.163234115	10.37856441	0.534923901	3.95E-18
HNRNPAB	44.660895	63.53667597	0.508577452	3.41E-15
NPM1	102.7340379	145.6744247	0.503833358	4.38E-13
PRC1	1.089103162	2.217932684	1.026074964	5.77E-15
CBX2	0.367085442	1.084481127	1.562817139	3.06E-13
YTHDF1	17.27808346	20.30924212	0.233193206	3.56E-09
YTHDF2	14.50239327	16.81944936	0.213839474	5.30E-09
TPX2	1.0347345	3.029874484	1.549997392	5.25E-17
TACC3	1.298188131	2.170450182	0.741494838	4.63E-08
TAZ	2.784283846	3.810698132	0.452749033	1.88E-06
SAFB	11.79808531	15.10502838	0.356476149	1.35E-11
FXR1	9.203646827	10.02964019	0.123992322	0.005602332
HOXA13	16.09981117	10.9955413	−0.550125139	3.32E-08
HOXD13	9.810809954	2.822284597	−1.797508771	2.38E-19
RUNX2	1.74434811	1.150934298	−0.599882501	4.03E-08
GATA3	13.37469968	3.252197009	−2.040019937	1.15E-19
G3BP1	11.10531135	12.69558022	0.193076491	0.000534024
GIT1	10.42461598	12.29219986	0.237748891	4.15E-06
PTPN11	20.451457	15.2610564	−0.422348795	6.74E-08
APC	2.995679231	2.649843384	−0.176976059	0.011275548
EBF1	1.465760533	1.025408614	−0.515450502	0.000213924
SMAD3	7.915414846	5.483742992	−0.529504006	1.47E-10
UBQLN4	12.08719142	13.36230088	0.144689391	0.007721401
FBL	50.33308096	63.29813385	0.330656057	0.000187085

USP42	2.167184885	2.695614113	0.314791748	4.15E-06
DYRK3	1.167097583	0.828177459	-0.49491335	1.68E-06
SURF6	9.266019673	11.71624694	0.338488855	2.23E-10
GNL2	7.417092308	10.96579009	0.564084133	1.45E-20
LAT	0.038749709	0.084402189	1.123094962	1.24E-08
POLR1E	10.36784648	9.822291972	-0.077984648	0.040738674
SPOP	20.95039562	15.12426634	-0.470112326	6.89E-11
POLR1B	3.051370731	4.038074333	0.404209995	1.87E-06
LMNA	43.90098192	34.68662434	-0.339873763	0.00049882
HSPB3	3.054227643	0.97424442	-1.648451927	3.59E-08
HSPB2	1.545118887	0.779845661	-0.986457315	1.71E-13
SFPQ	28.76837288	39.68394876	0.464071963	5.68E-15
NONO	57.57455769	76.38290844	0.407818432	6.24E-15
ZNF207	6.876997654	7.953007697	0.209721714	3.44E-06
PCM1	9.663241731	7.43755095	-0.377679605	2.24E-09
SRRM2	40.54898135	48.64652096	0.26267096	0.000645294
KEAP1	22.63129462	26.00638675	0.200546854	5.07E-07
MATR3	0.269134813	0.404358654	0.587306473	1.22E-07
MDC1	4.241852885	3.728441679	-0.186121811	0.000249648
SRSF1	38.93699231	47.72733994	0.293674484	1.87E-12
PTPN23	11.50051804	13.25812211	0.205177597	0.000302448
ERC1	6.119583058	4.008575694	-0.610343645	8.68E-12
TRIM28	58.38677673	75.01262006	0.361491665	1.09E-13
ORC1	0.307829366	0.469619194	0.60936051	0.000173295
ORC2	4.179730942	4.641900363	0.15130548	0.002489223
ORC5	7.431211058	9.77403285	0.395356609	4.44E-06
ORC3	6.996392077	6.223857585	-0.168802091	0.001129053
ORC6	0.248048843	0.521784212	1.072829062	4.30E-10
USH1G	0.102939085	0.028814465	-1.836925701	1.50E-15
MYO7B	0.179049707	0.130293431	-0.45859581	4.03E-05
USH1C	0.056394448	0.064074827	0.184204529	6.70E-05
TEAD1	9.527131462	4.972401405	-0.938099131	5.68E-15
UBAP2L	16.79264623	19.30446429	0.201104927	0.000168297
HSF1	15.26749942	19.57034234	0.358205202	1.02E-08
FMR1	4.921639058	4.365127012	-0.173115225	0.000575414
DCP1A	5.854174865	5.301707768	-0.14300869	0.017386154
PSMB2	17.68530596	19.32564574	0.127965442	0.013238359
RAD23B	39.09710404	50.79010683	0.377485759	2.28E-11
MORC3	6.528662769	4.990082831	-0.387723759	1.42E-07
MED16	16.14621792	20.60246678	0.351620816	8.95E-08
SRSF2	29.06144769	35.4873023	0.288196334	9.45E-09
BRD3	7.349646135	8.559949066	0.219927422	0.018540657
HDAC6	4.432055635	4.888437084	0.141397294	0.018909132
RBM20	0.214741174	0.136386816	-0.654894643	1.38E-08

STIL	0.533218835	1.181197993	1.147451164	7.97E-17
CBX5	8.542801904	7.889481866	-0.114778773	0.014346854
KMT5C	1.365486969	2.29891869	0.751539894	8.11E-14
HSPA5	184.22025	220.5371795	0.259590239	0.000914081
HSPA8	192.6662273	232.5317121	0.271323779	0.000448505
HSPA1L	1.704020894	1.359937228	-0.325402965	5.06E-06
ATR	2.123162188	2.640851289	0.31478848	0.002542465
DDX5	70.95189	83.52333293	0.235338168	0.000720597
NELFE	21.01806577	25.11752807	0.257064581	1.43E-05
NELFA	5.896556712	6.929042814	0.23278333	1.30E-06
GLE1	10.50909873	11.84417776	0.172539101	0.001265332
CAV1	52.29986996	16.21313432	-1.68964434	4.77E-20
TEAD4	2.915334365	3.347540157	0.199440004	0.001136355
ANKRD52	6.203313115	8.058423016	0.377458592	1.60E-05
AXIN1	4.433768981	6.181824305	0.47949905	7.04E-16
DACT1	3.709440508	2.518208912	-0.558803628	6.53E-06
PLRG1	9.326304731	8.50078613	-0.133709305	0.000720597
